# Comprehensive Analysis of Codon Usage Patterns in Chinese Porcine Circoviruses Based on Their Major Protein-Coding Sequences

**DOI:** 10.3390/v14010081

**Published:** 2022-01-03

**Authors:** Hua Feng, Joaquim Segalés, Fangyu Wang, Qianyue Jin, Aiping Wang, Gaiping Zhang, Giovanni Franzo

**Affiliations:** 1 Henan Provincial Key Laboratory of Animal Immunology, Henan Academy of Agricultural Sciences, Zhengzhou 450002, China; huafeng68@outlook.com (H.F.); sprinkle.w@126.com (F.W.); ubox3301@outlook.com (Q.J.); 2 OIE Collaborating Centre for the Research and Control of Emerging and Re-Emerging Swine Diseases in Europe (IRTA-CReSA), 08193 Cerdanyola del Vallè, Spain; joaquim.segales@irta.cat; 3 UAB, Centre de Recerca en Sanitat Animal (CReSA, IRTA-UAB), Campus de la Universitat Autònoma de Barcelona, 08193 Barcelona, Spain; 4 Departament de Sanitat i Anatomia Animals, Facultat de Veterinària, Universitat Autònoma de Barcelona, 08193 Barcelona, Spain; 5 School of Life Sciences, Zhengzhou University, Zhengzhou 450001, China; pingaw@126.com; 6 Department of Animal Medicine, Production and Health (MAPS), University of Padua, 35020 Legnaro, Italy; giovanni.franzo@unipd.it

**Keywords:** porcine circovirus, codon usage pattern, natural selection, mutation pressure, viral host adaptability

## Abstract

Porcine circoviruses (PCVs) are distributed in swine herds worldwide and represent a threat to the health of domestic pigs and the profits of the swine industry. Currently, four PCV species, including PCV-1, PCV-2, PCV-3 and PCV-4, have been identified in China. Considering the ubiquitous characteristic of PCVs, the new emerged PCV-4 and the large scale of swine breeding in China, an overall analysis on codon usage bias for Chinese PCV sequences was performed by using the major proteins coding sequences (ORF1 and ORF2) to better understand the relationship of these viruses with their host. The data from genome nucleotide frequency composition and relative synonymous codon usage (RSCU) analysis revealed an overrepresentation of AT pair and the existence of a certain codon usage bias in all PCVs. However, the values of an effective number of codons (ENC) revealed that the bias was of low magnitude. Principal component analysis, ENC-plot, parity rule two analysis and correlation analysis suggested that natural selection and mutation pressure were both involved in the shaping of the codon usage patterns of PCVs. However, a neutrality plot revealed a stronger effect of natural selection than mutation pressure on codon usage patterns. Good host adaptation was also shown by the codon adaptation index analysis for all these viruses. Interestingly, obtained data suggest that PCV-4 might be more adapted to its host compared to other PCVs. The present study obtained insights into the codon usage pattern of PCVs based on ORF1 and ORF2, which further helps the understanding the molecular evolution of these swine viruses.

## 1. Introduction

The name of Porcine circovirus (PCV) originally came from the first identification of a small circular single-stranded DNA virus identified in porcine kidney-15 (PK-15) cells (ATCC-CCL-33) in 1982 [1]. This virus was subsequently renamed as Porcine circovirus 1 (PCV-1) since the emergence of additional PCVs. Until now, besides PCV-1, three, other species of PCV have been identified subsequently, including Porcine circovirus 2 (PCV-2), Porcine circovirus 3 (PCV-3) and Porcine circovirus 4 (PCV-4) [2]. PCVs (at least PCV-1, PCV-2 and PCV-3) are considered ubiquitous in the pig population and represent a threat to the swine health and the development of global swine industry [3,4,5].

As a country with a long history of swine breeding, China has suffered huge economic losses due to PCV-2 infection and all PCV species have been identified in this country at different time-points [6]. Although PCV-1 was first identified in 1974 [1], no clear data on the start of the virus circulation was reported in China, probably as it was not investigated properly since it was considered non-pathogenic for swine [7,8]. PCV-2 drew enormous attention from the swine industry since its first identification in China in 1998 [6], being responsible for porcine circovirus diseases (PCVDs) [9]. Particularly, outbreaks of PCV-2 systemic disease (previously known as postweaning multisystemic wasting syndrome) since 2002 made this virus an economically significant agent for the Chinese swine industry [10]. Thereafter, PCV-3 was initially detected from the tissue samples from a U.S. affected farm with reproductive failure and cases of porcine dermatitis and nephropathy syndrome (PDNS) by using high-throughput sequencing method in 2016 [11,12] and was also reported in China in 2018 [13]. Similar to PCV-2, the high prevalence of PCV-3 in pig herds also suggested its high infectiousness [3]. Very recently, PCV-4 was firstly identified in China in pigs with severe clinical disease but also in subclinical subjects [14], and the data also showed that this virus could infect piglets of different ages. However, the information on PCV-4 is still limited, and more studies are needed to further characterize this new circovirus.

As other Circoviridae family members, PCVs are small (about 17 nm in diameter), non-enveloped viruses with a closed-circular single-stranded ambisense DNA genome [15,16,17]. The genome size for PCVs ranges from around 1.7 kb to 2 kb [3]. All PCVs show a similar genome organization, which include two main open reading frames (ORFs), ORF1 and ORF2, encoding the replicase and capsid proteins in an opposite direction, respectively. The proteins encoded by ORF1 and ORF2 are essential for virus replication, although the length and sequence of these two ORFs are significantly different among viral species; the length of ORF1 is about 939 nt, 945 nt and 891 nt for PCV-1, PCV-2 and PCV-4 [3]; for PCV-3, the length of ORF1 is not fully identified, since the location of its start codon is still not located, and the length of the identified part of PCV-3 ORF1 is around 891 nt [11,12]. On the other hand, the length of ORF2 is about 702 nt, 702 nt, 645 nt and 687 nt for PCV-1, PCV-2, PCV-3 and PCV-4, respectively. Despite such a simple genome, PCVs feature a complex interaction with the host. Therefore, it is intriguing to explore the characteristics and structure of PCV genomes and clarify if and how it could affect the interaction with the host.

The phenomenon of preferential usage of synonymous codons during translation is known as synonymous codon usage bias and has been widely identified in different species [18,19,20,21]. Since the life cycle of viruses is mostly dependent on their host, the study of codon usage bias of viruses could further clarify the relationship between viruses and their hosts, and also illuminate the molecular evolution and the virus gene regulation [22,23]. In the current study, a total of 1433 Chinese strains of different PCV species were selected, and their ORF1 and ORF2 codon usage patterns were comprehensively analyzed by a series of bioinformatic methods. The obtained results extended the understanding of synonymous codon usage patterns of different PCVs, and further clarified the molecular evolution and the host adaption of PCVs.

## 2. Materials and Methods

### 2.1. Sequence Selection

A total of 1999 complete genome sequences of PCVs retrieved from pigs in China were obtained from the NCBI GenBank database (https://www.ncbi.nlm.nih.gov/, accessed on 7 July 2021), (50 PCV-1, 1542 PCV-2, 377 PCV-3, 30 PCV-4). ORF1s and ORF2s of all these strains were extracted by MEGA-X program (version 10.1.8). After removing the stop codon of ORF1 and the start codon of ORF2, the extracted ORFs were concatenated in the following order: ORF1-ORF2 (ORF12), and for PCV-3, the identified part of ORF1 was employed. Duplicate sequences (i.e., those displaying 100% genetic identity) were removed. The remaining ORF12s were further analyzed for potential recombination by RPD4 software (*p* value cutoff at 0.05) using RPD, GENECONV, Chimaera, MaxChi, BootScan, SiScan and 3Seq algorithms [24]. The sequences with recombination events supported by at least one method were removed. After the distance and recombination analysis, 44 strains of PCV-1, 1304 of PCV-2, 357 of PCV-3 and 28 of PCV-4 were included in the final dataset (Table S1) and used to explore the codon usage patterns of PCVs.

### 2.2. Composition Analysis of the Selected PCV Strains

Nucleotide compositions of the selected sequences were analyzed by CAIcal (http://ppuigbo.me/programs/CAIcal/, accessed on 28 July 2021) [25] and CodonW software (version 1.4.4, written by John Peden, http://sourceforge.net/projects/codonw, accessed on 13 August 2021). The contents of the A, U, G, C and the frequencies of mononucleotide at the third codon position (A3, U3, G3, C3) of synonymous codons were calculated. The GC frequencies at the three codon positions (GC1, GC2, GC3) were explored separately. Additionally, the mean frequency of GC at the first and second codon sites was also computed.

### 2.3. Relative Synonymous Codon Usage (RSCU) Value and Principal Component Analysis (PCA) Analysis

The RSCU value represents the ratio between the observed frequency of one specific synonymous codon and the expected frequency, which was widely used to evaluate the codon usage bias of coding sequence. The codon with RSCU value = 1, or <1, or >1 indicate that no bias, negative codon usage bias, and positive codon usage bias, respectively. Furthermore, the synonymous codon is considered as over-represented or under-represented codons when the RSCU values are higher than 1.6 or lower than 0.6, respectively. In the current study, the RSCU values of the selected PCV sequences were calculated with CodonW software and the reference RSCU values of swine (*Sus scrofa*) were downloaded from the Codon and Codon Pair Usage Tables (CoCoPUTs) database [26].

PCA is a multivariate statistical method that can reduce the dimensions of the data to display the main variation trend. In this analysis, the RSCU values of all the selected sequences were arranged into a 59-dimensional vector based on 59 synonymous codons (excluding AUG, UGG and the three stop codons), and converted into unrelated factors (principal components). The first two components were selected as the two axes for PCA plots. PCA was performed in GraphPad Prism 9.

### 2.4. Effective Number of Codons (ENC) and ENC-Plot

The ENC value is used to evaluate the level of codon usage bias, which is an absolute measure ranging from 20 to 61. The lower the ENC value, the higher the degree of bias level. In general, the evaluated sequence was considered to have a high codon usage bias if ENC value was lower than 35. The ENC values of all sequences of the current study were calculated by CodonW software. ENC plot analysis can be drawn to display the relationship between ENC value and GC3 and demonstrate the factors influencing the codon usage variation. The expected ENC curve was drawn by the following formula:

$$ENC^{expect}=2+s+\left(\frac{29}{s^{2}+{\left(1-s\right)}^{2}}\right)$$

where “*s*” represents the GC3s values. If the relating points are distributed on or around the expected curve, the codon usage is only influenced by mutation pressure; otherwise, if the points lie below the curve, it is considered that other forces constrain the codon usage.

### 2.5. Parity Rule 2 (PR2) Analysis

Parity rule 2 (PR2) plot analysis was applied to clarify if the codon usage bias resulted from mutation or natural selection pressure. The AT bias [A3/(A3 + U3)] and GC bias [G3/(G3 + C3)] were used as ordinate and abscissa, respectively, to plot the PR2-biases. When calculated values lie in the plot center (0.5, 0.5), no bias between mutation pressure and natural selection is expected, since A3 = U3 and C3 = G3 at the center point.

### 2.6. Neutrality Plot Analysis

Neutrality plot analysis was performed to analyze and compare the extent of mutation and natural selection pressure by plotting GC12 against GC3 values. In the plot, each point represented one PCV sequence, and the regression line was also plotted. If all points fell on or near the diagonal line (slope = 1), it is considered that the mutation pressure is the main force shaping the codon usage pattern of PCV genes. On the other hand, if the regression line tended to run parallel with the x axis, natural selection plays a dominant role (slope = 0). From this perspective, the regression coefficient can be interpreted as a measure of the mutation–selection equilibrium.

### 2.7. Codon Adaptation Index (CAI) Analysis

CAI analysis was mainly used for explaining the codon usage bias due to the natural selection pressure induced by virus adaptation to the codon usage pattern of the host. CAI value ranges from 0 to 1; the higher the value, the better adaption to the host. In the current study, the CAI value of PCVs was obtained with CAIcal using a codon usage table of *Sus scrofa* as a reference (see Section 2.3 of Materials and Methods).

### 2.8. Hydropathicity and Aromaticity Analysis

General average hydropathicity (Gravy) and aromaticity (Aroma) were evaluated as two major factors influencing translation and natural selection, which were calculated by CodonW software. Briefly, the Gravy value is calculated as the sum of hydropathy values of all amino acids in a sequence divided by the number of residues, while the Aroma value is the relative frequency of aromatic amino acids in the considered amino acid sequence.

### 2.9. Correlation Analysis

Correlation analysis was applied between A3, T3, G3, C3, GC3, ENC and A%, T%, G%, C%, GC%, Gravy values and Aroma values by using the Spearman’s rank method of GraphPad Prism 9.

## 3. Results

### 3.1. Characteristics of Nucleotide Composition of PCVs

The nucleotide composition was analyzed to assess its impact on codon usage pattern. The results indicated that the overall distributions of A (28.01 ± 0.27%), U (23.35 ± 0.32%), C (24.46 ± 1.63%) and G (24.18 ± 1.72%) were unbalanced for all selected PCV ORF12 sequences and showed a higher AU bias (Table 1). For each PCV species (PCV-1-4), the frequency of A nucleotide was relatively higher than the others. The frequency of the four nucleotides followed the same trend for PCV-1 and PCV-2 (A% > C% > U% > G%), however they were different for PCV-3 (A% > G% > U% > C%) and PCV-4 (A% > G% > C% > U%). The analysis of the nucleotide at the third position of the codons showed C3 were most preferred for PCV-1 (29.99 ± 0.38), PCV-2 (30.12 ± 0.36) and PCV-4 (30.06 ± 0.19), and U3 for PCV-3 (27.46 ± 0.32). Although the GC analysis indicated that all PCV species were AU rich, higher GC3 contents were also seen in each of them, even compared with GC1, GC2 and GC12. Interestingly, GC content in PCV-4 was almost equal to AU (49.95% vs. 50.05%), and the corresponding GC3% reached 59.51%.

### 3.2. Characteristics of the Codon Usage Bias of PCVs

The RSCU values of all PCV ORF12 were analyzed and compared with the RSCU of their host (*Sus scrofa*) (Table 2) to explore why AU contents were enriched, and GC3 contents were high in these genes. Overall, as shown in Table 3, the eighteen preferred codons used by all four PCVs and their host were totally different, and there was only one preferred codon (CAC) shared by all viral species and the host. Obtained results implied a G/C ending preferred trend in PCVs, which corresponds to the high GC3 contents in these viruses. Furthermore, for the host, all eighteen viral preferred codons were ending with G (six) or C (twelve), and there were only eight (AUC, CCC, ACC, GCC, CAC, CAG, GAC, GGC), eight (CUG, CCC, ACC, UAC, CAC, CAG, GGC, UGC), five (AGC, ACC, CAC, AAC, GAG) and thirteen (UUC, CUG, AUC, GUG, AGC, CCC, GCC, CAC, CAG, AAC, AAG, GAC, GAG) preferred codons for PCV-1, PCV-2, PCV-3and PCV-4, respectively, also preferentially used by their host (*Sus scrofa*). Besides, there were only five, four, five, four over-represented codons in PCV-1, PCV-2, PCV-3 and PCV-4, respectively, as well as eleven, eleven, nine and thirteen under-represented ones in PCV-1, and four and eleven in PCV-2.

**Figure 1:**
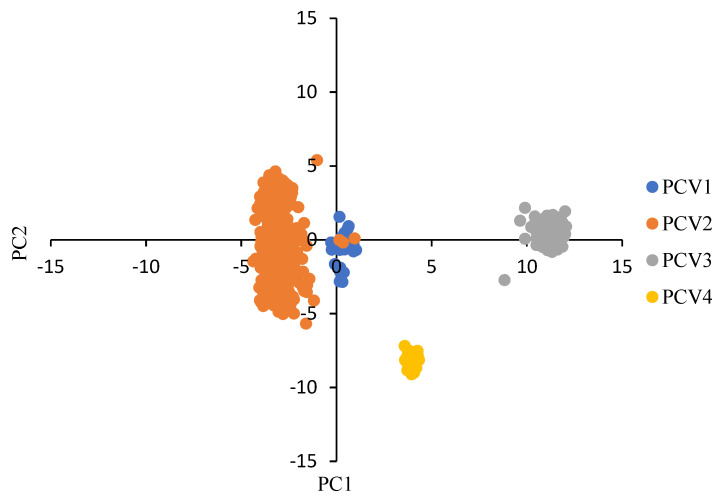
PCA of all selected PCVs based on the ORF12 RSCU values. PCV strains are plotted based on the first two principal components (PC) values. Each point represented one PCV strain. PCV-1, PCV-2, PCV-3 and PCV-4 were represented in blue, orange, grey and yellow, respectively.

As shown in Figure 1, the results of PCA indicated that the first and the second principal components (PC1 and PC2) accounted for 57.55% and 12.47% of total variation. All points were clearly distributed into four clusters, representing the four PCV species, although there was a certain overlap between PCV-1 and PCV-2.

### 3.3. Codon Usage Bias of PCVs Is Influenced Mainly by Natural Selection

**Figure 2:**
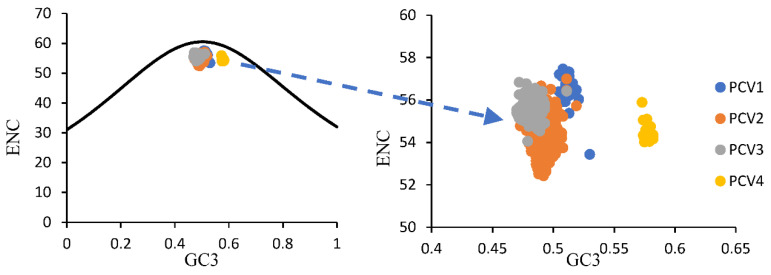
ENC-plot of ORF12s from different PCVs species. PCV-1, PCV-2, PCV-3 and PCV-4 were represented in blue, orange, grey and yellow, respectively. The black curve represented the expected ENC values based on GC3 composition only.

**Figure 3:**
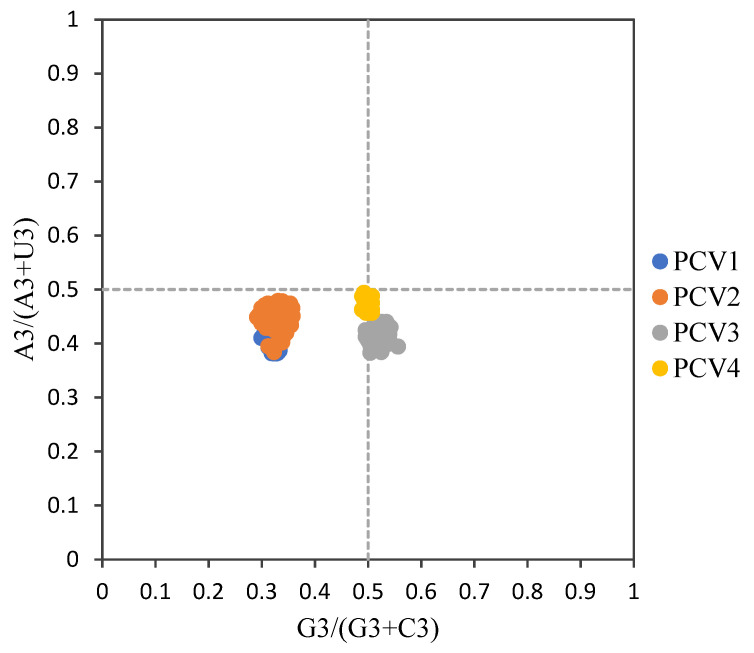
PR2 plot of ORF12s from different PCVs species. PCV-1, PCV-2, PCV-3 and PCV-4 were represented in blue, orange, grey and yellow, respectively.

For overall PCVs, the ENC values ranged from 52.42 to 57.46 (54.71 ± 0.87) and all values were higher than fifty, which implies a weak usage bias among all these four PCV species. Furthermore, the mean ENC values for PCV-1, PCV-2, PCV-3 and PCV-4 were 56.53 ± 0.87, 54.42 ± 0.75, 55.57 ± 0.39 and 54.48 ± 0.37, respectively. The ENC-plot (Figure 2) showed that all points were below the expected curve based on GC3 composition and formed four clusters based on PCV species. Furthermore, in the PR plot (Figure 3), all points for PCVs were located at the bottom of the plot, indicating an imbalance usage was found between A3, U3 and G3, C3, which further confirmed that the codon usage bias of PCVs is influenced by both natural selection and mutation pressure. The only partial exception is represented by PCV-4 since the points were located just below the plot center.

In addition, as shown in Table 4, significant positive correlations were observed between A and A3, C and C3, U and U3, G and G3, GC and GC3 in PCVs. Besides, significant correlations between ENC and each nucleotide/GC were also observed in different PCVs, especially in PCV-2 and PCV-3. These results indicated the existence of mutation pressure on codon usage bias. Gravy and Aroma values were considered as indicators of natural selection. In PCV-2, the Gravy and Aroma values were significantly correlated with A3, C3, U3, G3 and ENC, and the same significant correlations were also observed in PCV-3, excluding the Aroma and A3/G3 for this viral species. However, in PCV-1, significant correlations were only observed between Aroma and C3/ENC. In PCV-4, Gravy was only significantly correlated with C3 and Aroma was significantly correlated with T3, GC3 and ENC.

**Figure 4:**
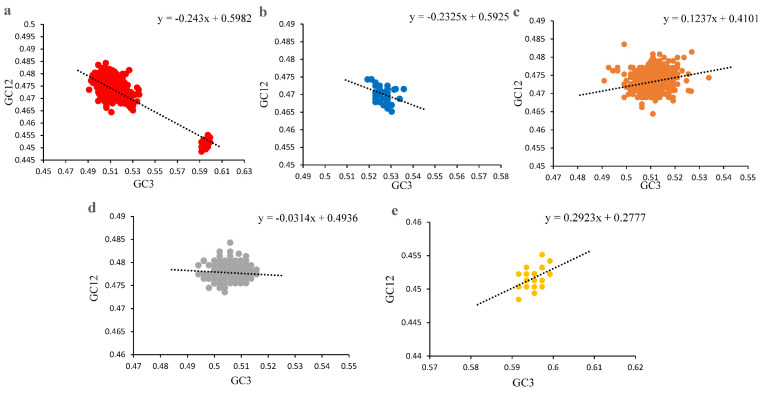
Neutrality plot analysis (NA) of ORF12s from different PCVs species. (**a**), NA of overall PCVs; (**b**), NA of PCV-1; (**c**), NA of PCV-2; (**d**), NA of PCV-3; (**e**), NA of PCV-4. The overall PCVs, PCV-1, PCV-2, PCV-3 and PCV-4 were represented in red, blue, orange, grey and yellow, respectively. The black dashed line represented the regression line for each PCV species; the regression equation is also reported.

The contribution of mutation pressure and natural selection was further tested by a neutrality plot (Figure 4) after removing the points with the outlier values. A slightly negative correlation was shown between GC3 and GC12 for all PCVs (R = −0.2204, *p* < 0.0001), and the slope was −0.243, which indicated that the natural selection from host plays a major role (75.7%) in the codon usage pattern of PCVs. In addition, further analysis for each of the species showed that for PCV-1, PCV-2, PCV-3 and PCV-4, the slopes were −0.2325, 0.1237, −0.0314 and 0.2923, and the contribution of natural selection were 76.75%, 87.63%, 96.85% and 70.77%, respectively. These results suggest that natural selection was the major factor during the forming process of PCV codon usage pattern.

### 3.4. Codon Adaptation Index Analysis

**Figure 5:**
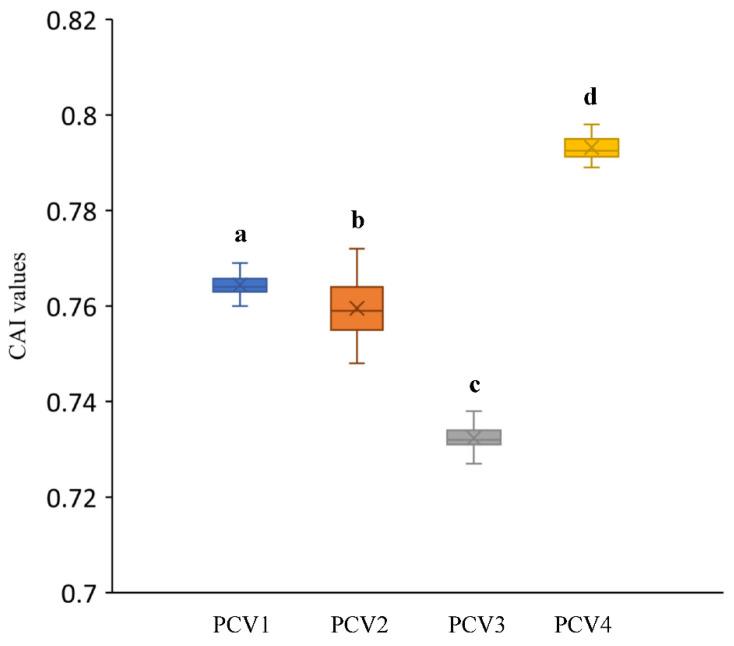
CAI values for each PCVs species. Different low-case letters represented statistically significant differences among different PCVs genotypes.

To explore the adaptation extent of PCVs in their hosts, the CAI values of all strains for each PCVs were analyzed by using the codon usage table of *Sus scrofa* as a reference. The CAI values calculated in the current study (Figure 5) were 0.764 ± 0.002 for PCV-1, 0.76 ± 0.005 for PCV-2, 0.732 ± 0.002 for PCV-3 and 0.786 ± 0.017 for PCV-4, respectively, and significant differences were also seen among these four groups by using the Mann–Whitney non-parametric test.

## 4. Discussion

Up to now, since the initial detection in 1974 as a picornavirus-like agent [27], PCV infections and the diseases associated with some of these viral species have been reported in the swine industry worldwide, seriously jeopardizing the health of domestic pigs and resulting in huge economic losses. The difference in the genome composition and codon usage patterns between PCVs and swine can influence the viral fitness, evolution, and the ability to replicate, transmit and eventually facilitate escape from the immune response [23,28,29]. This latter point is especially important for PCV-2 since vaccination may exert additional pressure on PCV-2 evolution [30], although it would be mainly related to particular epitope modifications. Therefore, although there are several previous studies focused on the codon usage bias of PCV-1, PCV-2 [31,32] and PCV-3 [33,34], with the emergence of PCV-4, a comprehensive analysis on the overall codon usage patterns of all PCV species allows a comparative evaluation of molecular evolution and host adaption of these viruses to their host, the pig.

Since the codon usage bias is typically more relevant in the highly expressed genes [35], ORF1 and ORF2, the two major protein-coding ORFs of PCVs, were selected and used to analyze codon usage patterns. In the current study, the data from composition analysis showed that the frequency of GC was lower than that of AU for all PCVs, however the frequency of GC3 was higher when compared with GC1 and GC2. Such a finding would imply that composition constraints played an important role in PCV codon usage bias. The results were consistent with the previous studies on PCV-2 [32] and PCV-3 [33], respectively. Furthermore, the frequencies of A3, U3, G3, C3 were very different from the overall frequencies of A, U, G, C regardless of the PCV species studied, which indicated that selection pressure may play a role during the formation of PCV codon usage pattern.

A large discrepancy in RSCU values between PCVs and their host indicated that several factors played a role in the PCV codon usage pattern. The RSCU values showed that, among 18 preferable codons, the number of GC ended codons was higher than AU end codons in PCV-1, PCV-2 and PCV-4, which mirrored the high GC3 frequency in these viruses. Whereas the number of preferred codons ending with AU were higher than the preferred codon ending with GC in PCV-3, the whole frequency of AU3 in PCV-3 was lower than that of GC3 (49.38% vs. 50.62%), indicating that the usage frequencies of codons ending with GC were higher than that of codons ending with AU. Interestingly, the number of preferred codons commonly used by PCV-4 and the host were higher than the other PCVs, implying that PCV-4 seems to be more adapted to the host, and therefore PCV-4 may have a higher potential to be efficiently translated than the other PCVs. Previous studies reported that the usage of preferred codons improves the translation efficiency; however, rare codons with low translation efficiency can also play a relevant biological role, for example facilitating the correct folding of viral proteins [22,23,36]. The low number of over- and under-represented codons in PCVs also indicated the existence of a low codon usage bias in these viruses, which could facilitate a certain plasticity in the viral replication in host/tissues featuring a different codon bias. On the other hand, compared with PCV-4, the lower number of commonly used preferred codons between PCV-1, PCV-2 PCV-3 and their host would also reflect that relatively higher level of natural selection was rendered on them than PCV4. Based on RSCU values, the variations in codon usage of all PCVs were explored by PCA, and the results showed that the codon usage patterns were different among PCVs, indicating that the contribution of the factors affecting the codon usage patterns was different among all PCV species. Besides, the point locations of PCV-3 were far away from that of the other PCVs, and the phenomenon was also observed in a previous report on the codon usage patterns of avian circoviruses, PCV-3 and other mammalian circoviruses including PCV-1 and PCV-2 [34], which could be explained by the occurrence of a combination of mammalian-virus rep genes with avian circovirus-like Cap genes in PCV-3 genome.

The ENC values for all PCV species fluctuated around 54.7 ± 0.871, indicating a low bias in these viruses, which may improve the replication of PCVs in their host by reducing the competition during translation. Additionally, the ENC-plot suggested that besides mutation pressure, other factors also affected the codon usage pattern of PCVs, which was consistent with previous reports [31,32,33]. The imbalanced frequency of A3, U3 and G3, C3 in PCVs indicated by PR plot suggested a relevant role of natural selection in shaping the codon usage pattern of the viruses. Besides, all the correlation results further confirmed that the mutation pressure and natural selection co-affected the formation of codon usage patterns for PCVs. The positive correlation between overall nucleotide compositions (A, C, U, G) and the ones at third codon position (A3, C3, U3, G3) indicated the role of the mutation pressure. This evidence is also confirmed by significant correlations between ENC and each nucleotide/GC. In addition, the correlations of both Gravy and Aroma values with the ENC value were significant for PCV-2 and PCV-3, which indicated the important role of natural selection in their codon usage patterns. On the other hand, for PCV-1 and PCV-4, the significant correlations were only observed between Aroma and ENC values, implying the existence of a weaker natural selection pressure compared with PCV-2 and PCV-3. Interestingly, the locations of the PCV-4 points in the PR plot were just below the center of the plot, which indicated that although natural selection and mutation pressure may play almost equal roles, the natural selection was still the main force affecting PCV4 codon usage pattern. The results were also reflected by the weak correlation of the tested factors in PCV-4.

Additionally, the data from the neutrality plot indicated the natural selection pressure was set as the main force for PCVs codon usage patterns, which was consistent with the conclusion from a previous study on PCV-3 [33]. Furthermore, the expanding swine breeding herds also set a strong selection pressure on the codon usage pattern of these viruses. The data also indicated that compared with PCV-1 and PCV-4, the natural selection seems stronger in the codon usage patterns in PCV-2 and PCV-3. However, another study using complete coding sequences of PCVs (49 PCV-1 and 46 PCV-2) from different parts of the world indicated that mutation pressure contributed more in PCV-1 codon usage bias, while mutation pressure and natural selection contributed equally in PCV-2 [31]. The different conclusion obtained in our study may be caused by the use of a different sequence of datasets as well as bioinformatic tests.

For all PCVs, CAI values >0.5 were demonstrated, which reflected a good adaptation for these viruses to their host. As shown in Figure 5, the highest CAI value of PCV-4 suggested a better adaption of this virus to the host compared to other PCVs, as it happens with other virus species [37]. This fact was also confirmed by the data obtained from the other analysis in the current study, such as the higher number of commonly used preferred codons, less bias on natural selection and mutation pressure, and relative lower natural selection pressure compared with the PCV-1, PCV-2 and PCV-3. However, the higher adaptability for PCV-1 and PCV-4 may lead to the competition of the translation resource with their host during protein translation and result in low translation efficiency, which may be a reason for the low prevalence of PCV-1 and PCV-4 [3,38,39]. A previous study on Crimean–Congo hemorrhagic fever virus also indicated that although the high CAI value represents a better adaption to the host, it exhibits a lower translation efficiency [22]. Whereas considering the high prevalence of PCV-2 and PCV-3, and the discrepancy regarding the RSCU of PCV-2 and PCV-3 with their host, the relative lower CAI value for PCV-2 and PCV-3 probably reflected that a relevant selection pressure on the codon usage pattern may contribute to the adjustment of the translation environment by using the codons that are not used as much by their host, and this finally benefits the viral replication and prevalence.

## 5. Conclusions

The current study gave an overall picture of codon usage patterns for Chinese PCVs by a series of bioinformatic methods. The obtained data indicated an overall weak usage bias among the four PCV species and showed that in addition to mutation pressure, natural selection played a major role in PCV codon usage. Interestingly, the data also indicated that PCV-4 may be more adapted to swine than the other PCVs, although it seems not to correlate with viral fitness and replication capability. Whereas considering the higher prevalence rate of PCV-2 and PCV-3, featured by lower CAI values, it can be speculated that a relevant selection pressure on the codon usage pattern is in place, which will benefit the viral replication and prevalence by adjusting the translation environment by employing the less used codons of their host. Besides, some inferences were also given in the current study, which needs to be confirmed by further studies. In summary, the obtained data in the current study contribute to the understanding of the evolution of whole PCVs and their host adaption.

## Figures and Tables

**Table 1:** Composition analysis of the selected sequences of PCVs.

Catalogs	PCV	PCV-1	PCV-2	PCV-3	PCV-4
A%	28.01 ± 0.27	27.47 ± 0.15	28.11 ± 0.17	27.67 ± 0.13	28.61 ± 0.11
C%	24.46 ± 1.64	25.58 ± 0.11	25.32 ± 0.19	21.36 ± 0.15	22.28 ± 0.12
U%	23.35 ± 0.32	23.61 ± 0.12	23.31 ± 0.17	23.61 ± 0.11	21.44 ± 0.09
G%	24.18 ± 1.72	23.34 ± 0.19	23.27 ± 0.14	27.37 ± 0.13	27.67 ± 0.07
A3%	21.84 ± 0.66	20.42 ± 0.40	21.95 ± 0.37	21.91 ± 0.37	17.95 ± 0.24
C3%	28.68 ± 2.83	29.99 ± 0.38	30.12 ± 0.36	23.17 ± 0.39	30.06 ± 0.19
U3%	26.98 ± 0.74	26.86 ± 0.26	26.94 ± 0.45	27.46 ± 0.32	22.55 ± 0.23
G3%	22.50 ± 2.77	22.73 ± 0.32	20.99 ± 0.34	27.45 ± 0.29	29.45 ± 0.18
GC%	48.65 ± 0.28	48.92 ± 0.20	48.59 ± 0.22	48.72 ± 0.17	49.95 ± 0.14
GC1%	50.15 ± 0.88	50.03 ± 0.37	50.59 ± 0.29	48.52 ± 0.19	50.67 ± 0.16
GC2%	44.61 ± 1.38	43.99 ± 0.25	44.07 ± 0.26	47.03 ± 0.25	39.65 ± 0.21
GC3%	51.18 ± 1.19	52.72 ± 0.44	51.10 ± 0.40	50.62 ± 0.45	59.51 ± 0.20
GC12%	47.38 ± 0.40	47.01 ± 0.23	47.33 ± 0.22	47.77 ± 0.14	45.16 ± 0.15

**Table 2:** RSCU values of different PCV species and its host *Sus scrofa*.

AA	Codons	PCV-1	PCV-2	PCV-3	PCV-4	*Susscrofa*
**Phe**	**UUU**	**1.13**	**1.15**	**1.08**	0.87	0.93
	**UUC**	0.87	0.85	0.92	**1.13**	**1.07**
**Leu**	**UUA**	0.27	0.16	0.38	0.18	0.49
	**UUG**	**1.64**	1.07	**1.54**	0.71	0.78
	**CUU**	1.25	1.25	0.59	0.87	0.81
	**CUC**	1.22	1.24	1.30	1.40	1.18
	**CUA**	0.65	0.77	0.76	0.51	0.39
	**CUG**	0.96	**1.51**	1.44	**2.33**	**2.35**
**Ile**	**AUU**	0.96	**1.19**	**2.01**	1.12	1.06
	**AUC**	**1.20**	1.01	0.14	**1.27**	**1.42**
	**AUA**	0.85	0.79	0.86	0.61	0.52
**Val**	**GUU**	1.14	0.82	**2.20**	0.41	0.73
	**GUC**	0.46	0.81	0.44	1.10	0.98
	**GUA**	**1.29**	**1.19**	0.62	0.33	0.45
	**GUG**	1.12	1.18	0.73	**2.15**	**1.83**
**Ser**	**UCU**	0.99	0.45	0.81	1.13	1.09
	**UCC**	**2.01**	**2.63**	1.24	1.03	1.30
	**UCA**	0.42	0.35	0.20	0.37	0.87
	**UCG**	0.19	0.01	0.93	0.00	0.37
	**AGU**	0.80	1.28	0.62	1.26	0.91
	**AGC**	1.59	1.27	**2.21**	**2.19**	**1.46**
**Pro**	**CCU**	0.98	0.94	1.03	0.34	1.12
	**CCC**	**1.68**	**1.60**	0.90	**1.53**	**1.31**
	**CCA**	1.02	1.12	**1.22**	1.36	1.06
	**CCG**	0.32	0.35	0.85	0.76	0.51
**Thr**	**ACU**	1.35	1.24	1.13	**1.58**	0.94
	**ACC**	**1.66**	**1.51**	**1.39**	1.28	**1.41**
	**ACA**	0.46	0.70	1.05	0.79	1.10
	**ACG**	0.52	0.55	0.43	0.35	0.55
**Ala**	**GCU**	1.57	**2.28**	**1.24**	1.00	1.02
	**GCC**	**1.59**	0.82	0.92	**1.55**	**1.64**
	**GCA**	0.42	0.42	0.64	0.55	0.89
	**GCG**	0.42	0.48	1.20	0.90	0.45
**Tyr**	**UAU**	**1.05**	0.93	**1.22**	**1.12**	0.87
	**UAC**	0.95	**1.07**	0.78	0.88	**1.13**
**His**	**CAU**	0.53	0.65	0.19	0.90	0.80
	**CAC**	**1.47**	**1.35**	**1.81**	**1.10**	**1.20**
**Gln**	**CAA**	0.73	0.84	**1.34**	0.93	0.50
	**CAG**	**1.27**	**1.16**	0.66	**1.07**	**1.50**
**Asn**	**AAU**	**1.23**	**1.39**	0.87	0.73	0.93
	**AAC**	0.77	0.61	**1.13**	**1.27**	**1.07**
**Lys**	**AAA**	**1.03**	**1.16**	**1.17**	0.90	0.87
	**AAG**	0.97	0.84	0.83	**1.10**	**1.13**
**Asp**	**GAU**	1.00	**1.06**	**1.22**	0.90	0.92
	**GAC**	**1.00**	0.94	0.78	**1.10**	**1.08**
**Glu**	**GAA**	**1.03**	**1.30**	0.90	0.74	0.86
	**GAG**	0.97	0.70	**1.10**	**1.26**	**1.14**
**Cys**	**UGU**	**1.12**	0.87	**1.14**	**1.46**	0.91
	**UGC**	0.88	**1.13**	0.86	0.54	**1.09**
**Arg**	**CGU**	0.67	0.51	0.76	0.57	0.45
	**CGC**	1.32	**1.93**	0.78	0.75	1.05
	**CGA**	0.28	0.36	0.36	0.00	0.67
	**CGG**	0.65	0.50	0.90	1.50	1.24
	**AGA**	**2.05**	1.83	**1.67**	**1.68**	1.29
	**AGG**	1.04	0.88	1.53	1.50	**1.30**
**Gly**	**GGU**	0.85	0.79	0.88	0.56	0.63
	**GGC**	**1.16**	**1.18**	0.57	1.15	**1.36**
	**GGA**	1.09	1.11	1.02	**1.33**	1.00
	**GGG**	0.91	0.92	**1.53**	0.96	1.01

The preferred codons for each PCV and *Sus scrofa* were marked in bold.

**Table 3:** The preferred codons used by PCVs and the ones commonly used by PCVs and Sus scrofa.

	Preferred Codons Ended with G/C	Preferred Codons Ended with A/U	Preferred Codons Commonly Used by PCVs and *Sus scrofa*
PCV1	AUC, UCC, CCC, ACC, GCC, CAC, GAC, GGC, UUG, CAG	GUA, AAA, AGA, GAA, UUU, UAU, AAU, UGU	AUC, CCC, ACC, GCC, CAC, CAG, GAC, GGC
PCV2	UCC, CCC, ACC, UAC, CAC, UGC, CGC, GGC, CUG, CAG	GUA, AAA, GAA, UUU, AUU, GUU, AAU, GAU	CUG, CCC, ACC, UAC, CAC, CAG, GGC, UGC
PCV3	AGC, ACC, CAC, AAC, UUG, GAG, GGG	CCA, CAA, AAA, AGA, UUU, AUU, GUU, GCU, UAU, GAU, UGU	AGC, ACC, CAC, AAC, GAG
PCV4	UUC, AUC, AGC, CCC, GCC, CAC, AAC, GAC, CUG, GUG, CAG, AAG, GAG	AGA, GGA, ACU, UAU, UGU	UUC, CUG, AUC, GUG, AGC, CCC, GCC, CAC, CAG, AAC, AAG, GAC, GAG

**Table 4:** The correlation between the content of A3, T3, G3, C3, GC3, ENC and A%, T, G, C, GC, Gravy values, Aroma values for each PCV genotype. (r values were shown in the table).

	A	C	U	G	GC	Gravy	Aroma
PCV-1-ORF12							
A3	0.52 **	0.13	−0.20	−0.36 *	−0.25	−0.22	−0.11
C3	0.13	0.74 **	−0.29	−0.33 *	0.10	0.12	0.37 *
U3	−0.20	−0.56 **	0.66 **	0.00	−0.30	−0.04	0.02
G3	−0.20	−0.46 **	0.04	0.42 **	0.13	0.09	−0.05
GC3	−0.17	0.34 *	−0.28	0.14	0.33 *	0.19	0.19
ENC	−0.30 *	−0.21	0.55 **	−0.06	−0.10	0.29	0.44 **
PCV-2-ORF12							
A3	0.41 **	0.35 **	−0.56 **	−0.42 **	0.06 *	−0.20 **	0.31 **
C3	−0.13 **	0.77 **	−0.56 **	−0.26 **	0.50 **	−0.13 **	0.27 **
U3	0.15 **	−0.74 **	0.87 **	−0.08 **	−0.73 **	0.18 **	−0.32 **
G3	−0.31 **	−0.37 **	0.21 **	0.68 **	0.09 **	0.08 **	−0.12 **
GC3	−0.53 **	0.53 **	−0.47 **	0.42 **	0.76 **	−0.06 *	0.18 **
ENC	0.24 **	−0.41 **	0.38 **	0.03	−0.38 **	0.29 **	−0.58 **
PCV-3-ORF12							
A3	0.78 **	−0.20 **	−0.07	−0.48 **	−0.55 **	−0.28 **	0.07
C3	−0.28 **	0.78 **	−0.55 **	−0.18 **	0.61 **	0.12 *	−0.11 *
U3	−0.10	−0.59 **	0.82 **	0.10	−0.48 **	0.18 **	0.11 *
G3	−0.43 **	−0.33 **	0.10	0.80 **	0.25 **	0.16 **	0.05
GC3	−0.57 **	0.57 **	−0.48 **	0.32 **	0.79 **	0.15 **	−0.11 *
ENC	0.03	0.37 **	−0.28 **	−0.25 **	0.19 **	−0.18 **	−0.11 *
PCV-4-ORF12							
A3	0.60 **	−0.18	−0.35	−0.21	−0.30	−0.05	0.15
C3	−0.46 *	0.73 **	−0.58 **	0.16	0.77 **	0.41 *	0.05
U3	−0.08	−0.47 *	0.88 **	−0.15	−0.48 *	−0.03	0.39 *
G3	0.09	−0.31	0.23	0.16	−0.21	−0.32	−0.34
GC3	−0.50 **	0.56 **	−0.43 *	0.39 *	0.71 **	0.17	−0.41 *
ENC	0.28	−0.08	0.13	−0.39 *	−0.22	−0.25	−0.42 *

* *p* < 0.05; ** *p* < 0.01.

## Data Availability

The data presented in this study are available in the current article and Supplementary Material.

## Supplementary Materials

The following are available online at https://www.mdpi.com/article/10.3390/v14010081/s1, Table S1: All PCV strains analyzed in the current study.
